# Comparing role of religion in perception of the COVID-19 vaccines in Africa and Asia Pacific

**DOI:** 10.1038/s43856-024-00628-2

**Published:** 2024-10-24

**Authors:** Shihui Jin, Alex R. Cook, Robert Kanwagi, Heidi J. Larson, Leesa Lin

**Affiliations:** 1 Saw Swee Hock School of Public Health, National University of Singapore and National University Health System, Singapore, Singapore; 2 Department of Statistics and Data Science, National University of Singapore, Singapore, Singapore; 3 Department of Infectious Disease Epidemiology, London School of Hygiene & Tropical Medicine, London, UK; 4 Centre for the Evaluation of Vaccination, Vaccine & Infectious Disease Institute, University of Antwerp, Antwerp, Belgium; 5 Department of Health Metrics Sciences, University of Washington, Seattle, WA USA; 6 Laboratory of Data Discovery for Health (D24H), Hong Kong Science Park, Hong Kong SAR, China; 7 WHO Collaborating Centre for Infectious Disease Epidemiology and Control, School of Public Health, LKS Faculty of Medicine, The University of Hong Kong, Hong Kong SAR, China

**Keywords:** Epidemiology, Viral infection

## Abstract

**Background:**

In the midst of the global COVID-19 vaccine distribution challenge, religion stands out as a key determinant of vaccine hesitancy and health choices. Notably, the multifaceted religious environments of Africa and the Asia Pacific remain under-researched in this context.

**Methods:**

Utilizing data from two survey waves conducted between 2021 and 2022, this cross-sectional study investigated the effects of religious beliefs on perceptions of compatibility between religion and vaccines and COVID-19 vaccine acceptance in Africa and Asia Pacific. Logistic regression models were employed, with interaction terms between socio-economic factors incorporated to account for variations among diverse subpopulations.

**Results:**

Among the eight religious groups identified, Atheists and Buddhists in the Asia Pacific exhibit the lowest agreement, with fewer than 60% acknowledging the religious compatibility of vaccines. Willingness to accept vaccines, however, is consistently higher in Asia Pacific by at least four percentage points compared to Africa, with the disparity widening further in the second wave. Impacts of education on vaccine perceptions vary across religious groups, while acknowledging vaccine compatibility with religion positively contributed to vaccine acceptance. Dynamics between region, religion, and other socio-demographic factors have changed substantially over time. All but Atheists and Muslims exhibit a higher propensity to endorse vaccines during Survey Wave 2.

**Conclusions:**

Our study reveals complex, context-dependent connections between vaccine attitudes and religion and the heterogeneous effects of time and education among different religious affiliations. Understanding the underlying drivers of these temporal variations helps inform tailored approaches aimed at addressing vaccine hesitancy, promoting vaccine uptake, and improving the well-being of each religious group.

## Plain Language Summary

This study examined the effects of religious beliefs on thoughts about agreement between religion and vaccines, and COVID-19 vaccine acceptance in Africa and Asia Pacific. Data came from surveys of individuals across many regions during the COVID-19 pandemic in 2021 and 2022. We found lower agreement to compatibility between vaccine and religious belief among Atheists and Buddhists in the Asia Pacific, while Africans were generally less likely to accept the COVID-19 vaccines. In addition, education influenced vaccine views differently across religious groups, and those who felt vaccination was compatible with their religion were more likely to accept a vaccine. These findings show we should monitor vaccine confidence and tailor efforts to reduce vaccine hesitancy among different subgroups of people.

## Abstract (web summary)

Jin et al. explore the role of religion in perception of COVID-19 vaccination across Africa and Asia Pacific regions. They highlight important similarities and differences that may help inform future public health interventions around preventative medicine.

## Introduction

Remarkable progress has been made in developing vaccines targeting coronavirus disease-2019 (COVID-19), with over ten licensed for human use as of June 2023 and many more in advanced stages of clinical development^1,2^. Despite their limited efficacy in preventing infections and the inevitable decline in vaccine-induced immunity over time, these COVID-19 vaccines have demonstrated great success in reducing the severity of COVID-19 and mitigating the adverse impacts of COVID-19 on human well-being^3,4^.

Large-scale vaccination campaigns have been launched in many countries since December 2020, when COVID-19 vaccines were first administered beyond clinical trial settings^3^. Although, in principle, the COVID-19 vaccines are universally available free of charge around the world^5^, the unequal access to vaccines across various regions remains a major barrier to population-wide immunization^6^. For instance, in the Asia Pacific region, where middle- and high-income countries are predominant, over 80% of the population had received at least one dose of COVID-19 vaccines by mid-2023. In contrast, the corresponding figure was less than 40% for Africa, which comprises a majority of lower-middle and low-income countries^7,8^.

Another key obstacle, however, is vaccine hesitancy, referring to the delay or rejection in accepting vaccines despite their availability^9^. This earlier definition has since been debated and, more recently, framed as a state of indecision^10^. Previous research has extensively examined contributors to vaccine hesitancy, which are often subject to specific circumstances but primarily encompass factors regarding accessibility of vaccines, individual perceptions of the disease’s risk, as well as confidence in both the vaccines themselves and the authorities responsible for vaccine distribution^9,11^. These subjective perceptions are further driven by socio-demographic factors, such as gender, education received, and religion^2,12^.

The strong association between religiosity and reduced support for vaccination has been well-documented in the literature. Vaccination may conflict with certain religious teachings^13^, such as the Muslim prohibitions of pork, such as the potential inclusion of pork derivatives (e.g., porcine), which would pose a dilemma for Muslims^14^. In other cases, misinformation about diseases and vaccines often circulates within religious contexts, influencing followers to have doubts about the safety and effectiveness of vaccines^15^.

Most of the studies on influences of religiosity target specific countries^16,17^, which exhibit notable differences in religiosity. However, few have made comparisons across diverse religions within a single region or between different regions. Christians and Muslims comprise the majority of the African population^18^, while religious beliefs in Asia Pacific tend to be more heterogeneous, with religions such as Buddhism and Hinduism being widely followed in addition to Christianity and Islam^19^.

In this study, we focus on people’s sentiments towards the COVID-19 vaccines at different time points, utilizing responses from two waves of surveys conducted in Africa and the Asia Pacific following the roll-out of the COVID-19 vaccines. Specifically, we explore the diversity of perceptions regarding the compatibility of vaccines with religious beliefs and acceptance of the COVID-19 vaccines across various religious groups and between the two waves. Additionally, we investigate the potential interplay between region, religion, and other socio-demographic factors, quantifying their collective impacts on individuals’ attitudes towards vaccines, as well as the temporal changes in these effects. The results demonstrate a close relationship between religious affiliation and vaccine perception, while also showing variations in the attitudes across space and time, as well as other socio-demographic subgroups. They highlight the need for targeted intervention measures to boost vaccine confidence and improve the overall immunization rates.

## Methods

### Data sources

Between mid-2021 and 2022, the Vaccine Confidence Project^TM^ ^20^ conducted several waves of comprehensive surveys to assess global public opinions about COVID-19 and vaccines. The data for this study encompass two survey waves across a total of 15 countries in Africa and the Asia Pacific region. During each wave, ~1000 adult residents from each country participated in the survey, providing their responses through telephone, face-to-face, or online interviews (Supplementary Information [SI] Table S1). The timing of the surveys differed in these two regions. Specifically, while the two waves in Africa took place around February and August 2022, those in Asia Pacific mainly occurred in June 2021 and May 2022 (SI Table S2). All the surveys were conducted following the roll-out of COVID-19 vaccination^5^, though the low vaccination rates in some African countries in 2022 may reflect the under-supply of COVID-19 vaccines (SI Table S4). More detailed survey information, including sampling approaches and languages used in each county, along with the roll-out of COVID-19 vaccines at the time of the surveys, can be found in Section 1 of the Supplementary Information.

### Selection of the study subjects

Aiming at quantifying the relationship between religious beliefs and people’s perceptions towards vaccines, our study focused on religions with over 50 believers among the respondents in Africa or Asia Pacific. The religions include Christianity and Islam in both regions, along with four more—Animism, Atheism, Buddhism, and Hinduism—in Asia Pacific. Respondents sharing the same religious beliefs within their respective region were grouped together as a single category, while those from different regions were assigned to distinct categories, even if they held the same religious beliefs. This generated a total of eight distinct religious groups. We also excluded from our study people who did not provide complete demographic information (age, gender, education received), identified as genders other than male or female (because they were too few in number to analyse), or refused to respond to the statement regarding religious compatibility of the vaccines, constituting approximately 1% of the total samples (SI Table S3). Such selections led to a final sample size of 14,121 and 14,107 for Wave 1 and 2, respectively, with roughly 48% of the respondents residing in Africa (Table 1, Supplementary Data 1).

**Table 1:** Composition (%) of respondents by religious belief and region in Wave 1 and 2 of the surveys

	Wave 1	Wave 2
Christian (Africa)	34	35
Muslim (Africa)	15	13
Animist (AP)	1.8	1.8
Atheist (AP)	11	10
Buddhist (AP)	24	25
Christian (AP)	11	10
Hindu (AP)	0.5	0.7
Muslim (AP)	4.4	4.3

AP stands for Asia Pacific.

The respondents’ attitudes towards vaccines were measured from two aspects: their perception of the compatibility of vaccines with their religious beliefs and their acceptance of the COVID-19 vaccines. Those supporting religious compatibility responded positively, by answering “tend to agree” or “strongly agree”, to the statement “Vaccines are compatible with my religious beliefs”, while those who accepted the COVID-19 vaccines had either been vaccinated against COVID-19 or endorsed the COVID-19 vaccines if they would help themselves, their families, friends or at-risk groups (SI Table S6).

### Assessment of impacts of religion

We began by estimating the proportions of people supporting religious compatibility and accepting the COVID-19 vaccines for different religious groups at the two-time points (i.e., Wave 1 and 2). We then quantified the influences of religion (short for ‘religion by region’, or ‘religious group’) using three multivariate logistic regression models:

- **Compatibility**: compatible ~ time + religion + education + gender + age,
- **Acceptance**: acceptance ~ time + religion + education + gender + age,
- **Acceptance with compatibility**: ${{\rm{acceptance}}}\, \sim \,{{\rm{time}}}+{{\rm{religion}}}+{{\rm{education}}}\,+\,{{\rm{gender}}}+{{\rm{age}}}+{{\rm{compatible}}}$,

where ${{{\rm{compatible}}}}$ is a binary variable which equals 1 if and only if the respondent exhibited a positive attitude towards the statement regarding compatibility between vaccines and his or her religious belief. ${Time}$ is also set as binary, with a value of 1 representing survey Wave 2. Nevertheless, in a subsequent sensitivity analysis, we tested this setting.

To account for the potential variations in the effects of religions across different subpopulations and time, we further constructed two logistic regression models that incorporated interactions between diverse socio-demographic factors, including religion, education, gender, and age. We initially considered full models with interaction terms between all these factors and thereafter removed terms whose estimated coefficients were not statistically significant. The final models we used for inference are:

I. **Compatibility**: compatible ~ religion × time × education + gender × age,
II. **Acceptance**: acceptance ~ religion × time × education + gender × age,
  A third model was employed to probe the link between COVID-19 vaccine acceptance and religious compatibility across different time points and religious groups, while accounting for the impacts of various socio-demographic covariates. The selection of interaction terms included in the model was similar to that for the previous two models. This analysis also served to highlight the disparities in impacts of religions between supporters and sceptics of religious compatibility of vaccines:
III. **Acceptance with compatibility**: ${{\rm{acceptance}}}\, \sim \,{{\rm{religion}}}\times {{\rm{time}}}\times {{\rm{compatibility}}}\,+{{\rm{religion}}}\times {{\rm{time}}}\times {{\rm{education}}}\,+\,{{\rm{gender}}}\times {{\rm{age}}}+{{\rm{age}}}\times {{\rm{compatible}}}$.

In addition, we also conducted some univariate and bivariate regression analyses to facilitate evaluating the average effects of factors of interest. Further details of the models and estimation methods are elaborated in Section 4 of the Supplementary Information (SI Table S7).

### Statistics and reproducibility

All the analyses and visualization were conducted with R^21^, using the survey (for quantitative analyses accounting for sample weights) and grid packages^22,23^.

### Ethical approval

The surveys conducted in Africa received ethical approval from the Human Research Ethics Committee of the University of Hong Kong (EA230420), while those in Asia Pacific were approved by the Institutional Review Board at the London School of Hygiene and Tropical Medicine (LSHTM 26636).

## Results

The religious compositions among the selected respondents differed greatly between Africa and Asia Pacific. Approximately 30% of the Africans identified as Muslims, with the remaining majority being Christians. In contrast, Buddhists constituted the largest proportion of Asia Pacific, comprising roughly a quarter of the population. Other prevalent religions in the region include Christianity and Atheism, each accounting for ~10% of the population. These religious compositions remained consistent across the two survey waves in both Africa and the Asia Pacific (Supplementary Data 1).

**Fig. 1:**
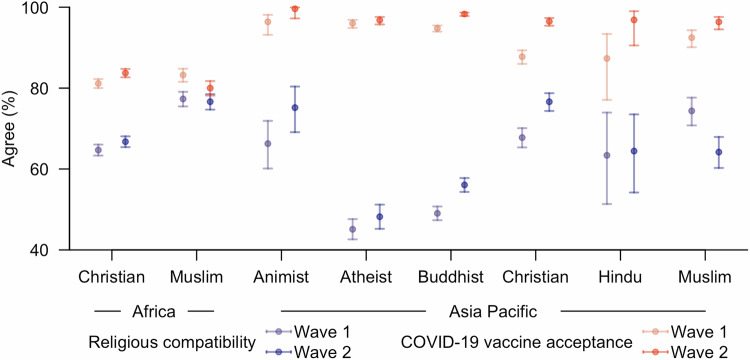
Endorsing rates in different by region and religion. Estimated proportion (point estimate and 95% confidence interval [CI]) in each religious group supporting religious compatibility or accepting the COVID-19 vaccines in Wave 1 and 2 of the surveys.

While survey results for both waves showed that people were, in general, more likely to accept the COVID-19 vaccines than to believe vaccines were compatible with their religious beliefs, the proportions varied greatly across different religious groups (Fig. 1, SI Table S8). Furthermore, a weak, positive correlation was observed between perception of religious compatibility and acceptance of the COVID-19 vaccines among all religious groups (SI Table S9).

### Compatibility of vaccines with religious beliefs

Compared to African Christians, the largest religious group in both waves of surveys, Muslims in both Africa and Asia Pacific were more likely to believe that vaccines were compatible with their religious beliefs during Wave 1. In the second wave, while African Muslims continued to display a stronger inclination towards vaccine compatibility with their faith than their Christian counterparts, in the Asia Pacific, the more supportive groups shifted to Animists and Christians.

**Fig. 2:**
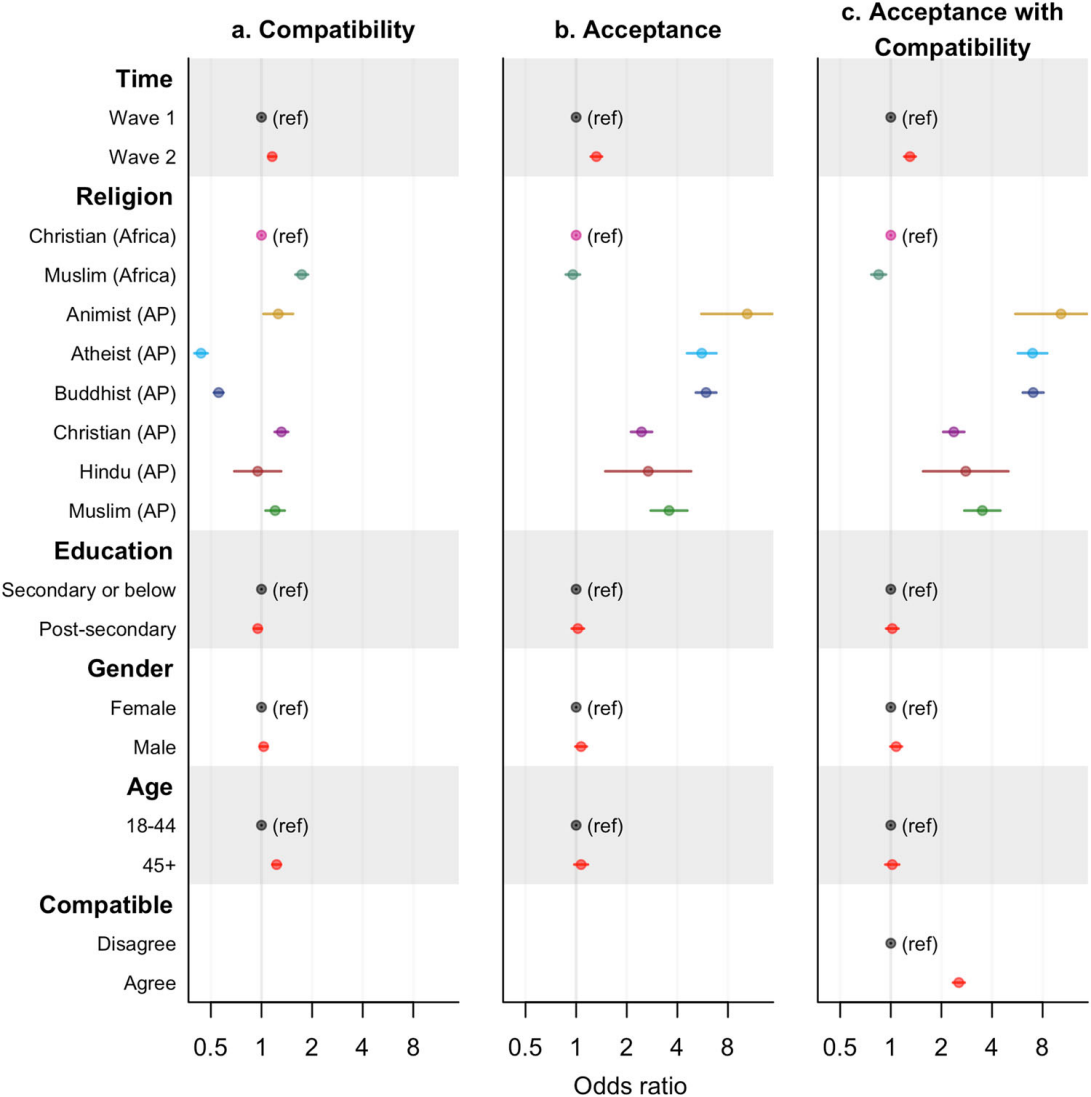
Estimated odds ratios (mean and 95% CI) for supporting religious compatibility of vaccines or accepting the COVID-19 vaccines. **a**–**c** correspond to the three established logistic regression models, with binary responses indicating either acknowledgement of compatibility between vaccines and religious beliefs (**a**) or acceptance of the COVID-19 vaccines (**b,** **c**). AP stands for Asia Pacific. Please note the logarithmic scales.

By contrast, Atheists and Buddhists in Asia Pacific exhibited significantly lower levels of support in both waves (Figs. 1–2, SI Table S10–11, Supplementary Data 2). This pattern remained consistent across different Asia Pacific countries, albeit with varying magnitudes (SI Figure S4). Furthermore, while Atheists were more likely to disagree with the compatibility between religions and vaccines (odd ratios [OR] and 95% confidence intervals [CI]: 1.11 [0.99–1.25] and 1.66 [1.47–1.89] for the two waves respectively), an even greater proportion found it challenging to formulate a response to this issue compared to other religious groups (ORs and 95% CIs: 3.65 [3.19–4.17] and 2.35 [1.96–2.81] for the two waves respectively) (SI Figure S5).

**Fig. 3:**
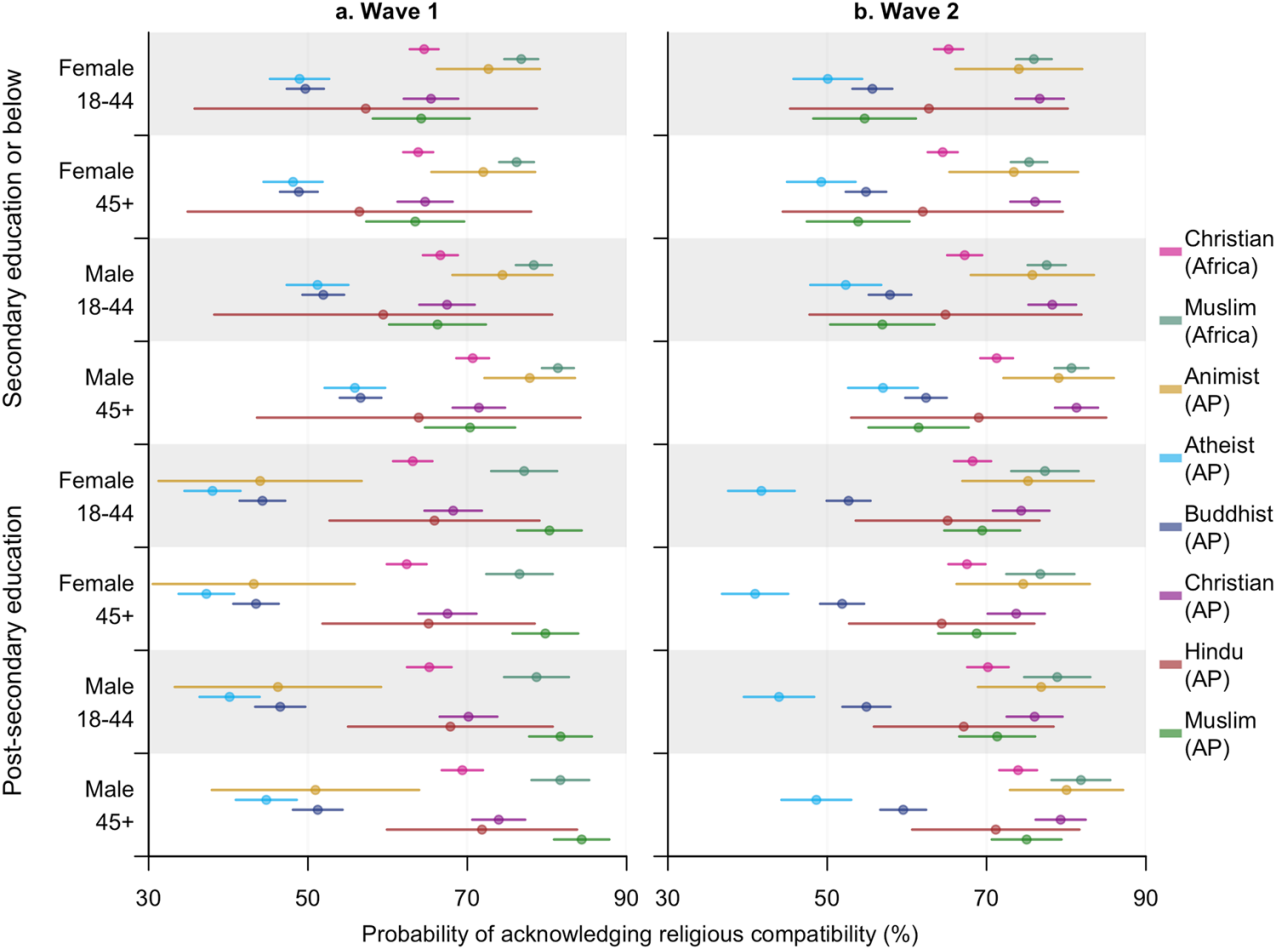
Estimated probabilities (mean and 95% CI) of supporting compatibility between vaccines and religious beliefs within diverse subpopulations at different time points. **a,** **b** represent the estimates for Wave 1 and 2, respectively. These probabilities were predicted from model **Compatibility** (i) by specifying the characteristics—religion (region-specific), age, gender, education received, and time surveyed—of the subpopulations. AP stands for Asia Pacific.

Nevertheless, significantly more people agreed with the religious compatibility of vaccines during Wave 2 of the surveys for all but African Muslims, as well as Atheists, Hindus, and Muslims in the Asia Pacific region (Table S13). The increase among Buddhists and Christians in Asia Pacific was greater than that among African Christians, but for Christians in Asia Pacific the growth was mainly attributed to the group with the most secondary school education (Fig. 3, S1, Table S13).

In addition to growth rates, education also made a difference in the relationship between religion and the perception of religious compatibility at a specific time point. A typical example was Animists in Asia Pacific in Wave 1, whose support towards religious compatibility was comparable to that of African Christians, but the odds ratio for accepting religious compatibility was significantly higher among people whose education levels were at most secondary school than African Christians with the same education background (OR: 1.45, 95% CI: 1.05–2.02) or Animists in Asia Pacific with higher levels of education (OR: 1.96, 95% CI: 1.51–2.56). In the Asia Pacific region, Buddhists constituted another religious demographic displaying similar lower levels of support among individuals who had attained post-secondary education, with odds ratios of 0.61 (95% CI: 0.45–0.83) and 0.37 (95% CI: 0.21–0.64) when compared to their less-educated counterparts during the two waves respectively. By contrast, more education promoted the support for religious compatibility among Muslims in the Asia Pacific (Fig. 3, SI Figure S1, Table S13), particularly during Wave 1 of the surveys (OR: 2.46, 95% CI: 1.34–4.52).

We also found higher supporting rates of religious compatibility with vaccines among people over 45 and larger gender difference for this subpopulation in the odds ratio of supporting religious compatibility, with males being more supportive (Figs. 2–3, SI Table S10, Supplementary Data 2).

### Acceptance of the COVID-19 vaccines

The vaccine acceptance rates were significantly higher among religious groups from Asia Pacific than Africa for both waves, while a prominent increase in inclination to accept the COVID-19 vaccines was observed among all but African Muslims and Atheists in Asia Pacific (Fig. 2, SI Table S10–11, Supplementary Data 2). The increase rates were also found to be larger among Buddhists, Christians, and Muslims in Asia Pacific than among African Christians, probably due to the longer temporal gaps between the two waves of the surveys in Asia Pacific countries (Table S2). African Muslims, however, showed a small yet non-trivial reduction in willingness to accept the COVID-19 vaccines (OR: 0.81, 95% CI: 0.69–0.95) (SI Table S8, S11).

**Fig. 4:**
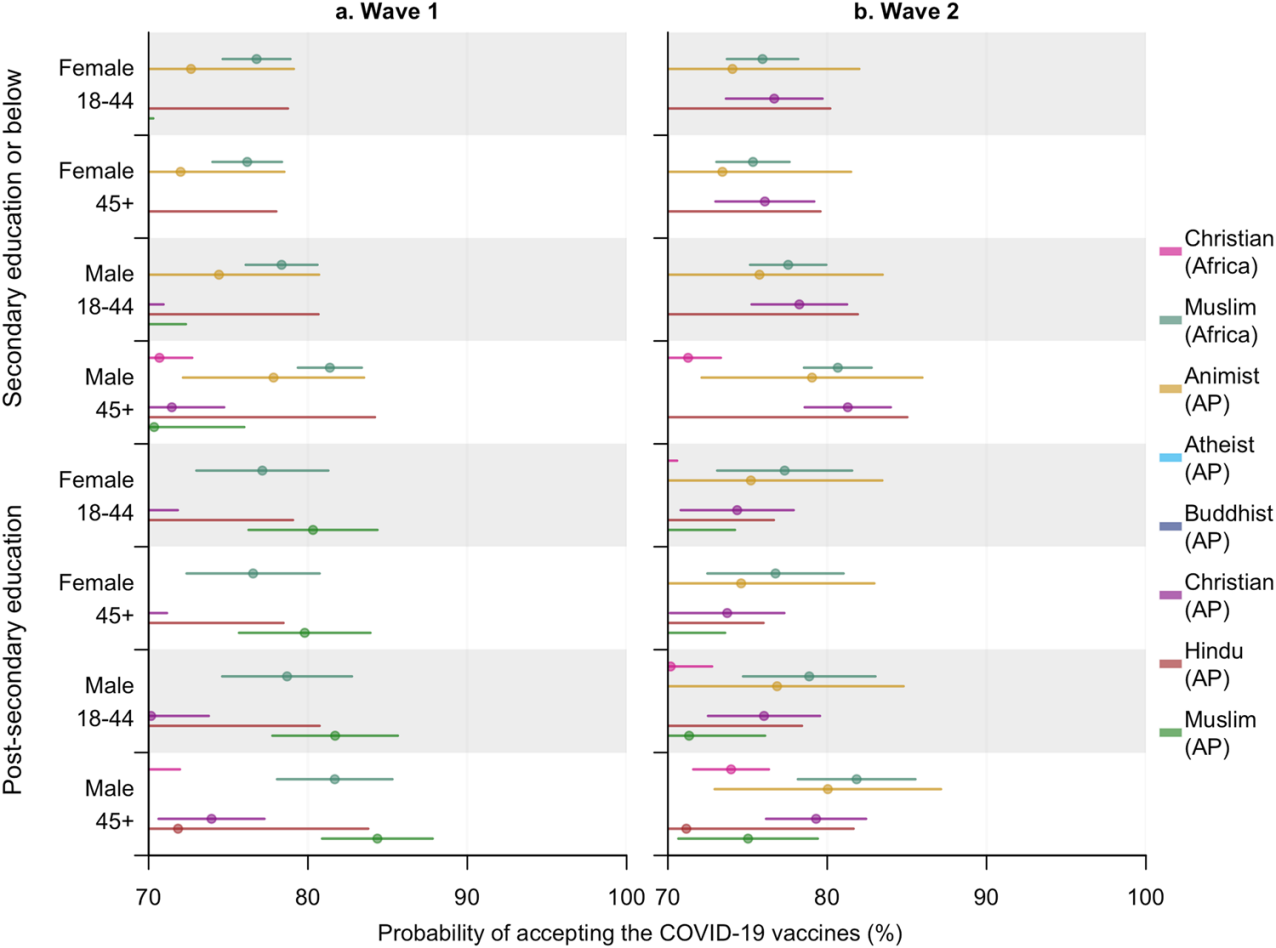
Estimated probabilities (mean and 95% CI) of accepting the COVID-19 vaccines within diverse subpopulations at different time points. **a,** **b** represent the estimates for Wave 1 and 2, respectively. These probabilities were predicted from model **Acceptance** (ii) by specifying the characteristics—religion (region-specific), age, gender, education received, and time surveyed—of the subpopulations. Part of the predictions for Animists in Wave 2 were not evident in the plot since almost all the respondents from this religious group were pro-vaccine, making it challenging to quantify the exact effects of the influencing factors. Accepting the vaccines means the respondent had been either vaccinated or willing to accept the COVID-19 vaccines for him or herself. AP stands for Asia Pacific.

Among groups with increased vaccine acceptance rates in Wave 2, people having received secondary school education or below generally contributed more to the growth compared to those with post-secondary education, with the only exception being Animists in Asia Pacific, among which only one respondent did not accept the COVID-19 vaccines. Particularly, despite a growth being observed among African Christians and Muslims in Asia Pacific as a whole, the between-wave difference was not evident among individuals with post-secondary education in either group (ORs and 95% CIs: 1.16 [0.97–1.38] and 2.11 [0.94–4.74], respectively). For Muslims in Africa, however, a decrease in willingness to accept the COVID-19 vaccines was observed across both education levels, with that among the more educated slightly larger (Fig. 4).

Furthermore, we also found great disparities in effects of education on the COVID-19 vaccine acceptance among the eight religious groups. More education (i.e., post-secondary) boosted individuals’ willingness to accept the COVID-19 vaccines for Christians in Asia Pacific and Muslims in both regions during Wave 1, while people with less education among Buddhists tended to be more pro vaccine during both waves (SI Fig. S2, Supplementary Data 2).

### Role of acknowledging religious compatibility in accepting the COVID-19 vaccines

**Fig. 5:**
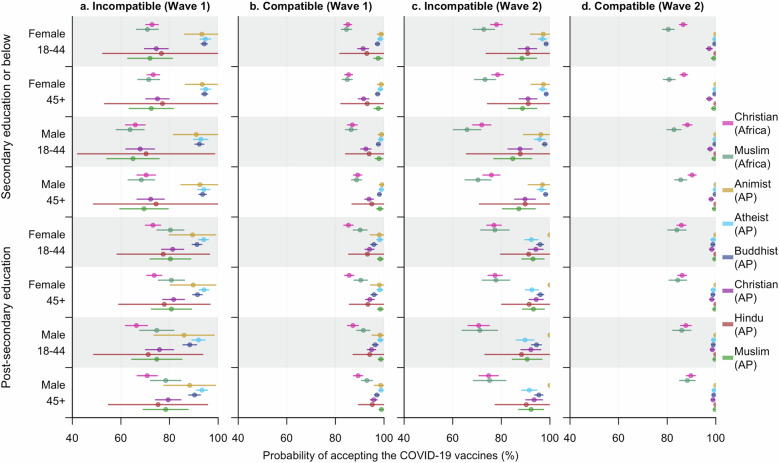
Estimated probabilities (mean and 95% CI) of accepting the COVID-19 vaccines within diverse subpopulations at different time points. **a,** **b** represent the estimates for individuals acknowledging and rejecting compatibility between vaccines and religious beliefs in Wave 1, while **c,** **d** correspond to Wave 2. These probabilities were predicted from model **Acceptance with Compatibility** (iii) by specifying the characteristics—religion (region- specific), age, gender, education received, time surveyed, and whether to agree with religious compatibility of vaccines—of the subpopulations. Part of the predictions for Animists and Hindus in Wave 2 were not evident in the plot since almost all the respondents in these two religious groups were pro-vaccine, making it challenging to quantify the exact effects of the influencing factors. Accepting the vaccines means the respondent had been either vaccinated or willing to accept the COVID-19 vaccines for him or herself. Compatible and Incompatible, respectively, refer to subgroups who did and did not think vaccines were compatible with their religious groups. AP stands for Asia Pacific.

In general, the perception of the compatibility of vaccines with their religious beliefs promoted the acceptance of the COVID-19 vaccines (Fig. 2, SI Table S10). Compared to African Christians, the effect of acknowledging this compatibility of religious beliefs and vaccine sentiments was more prominent among Christians and Muslims in Asia Pacific during Wave 1 of the surveys. During Wave 2, however, the influence was much weaker in Africa compared to that in any of the religious groups in Asia Pacific (Fig. 5, SI Tables S12 and S14). Moreover, while people skeptical of the compatibility of religious beliefs with vaccines did not exhibit a reduced willingness to accept the COVID-19 vaccines during Wave 2, a notable decrease was found among people who agreed with the compatibility of vaccines and their religious beliefs and belonged to the subgroup displaying more vaccine hesitancy, i.e., African Muslims (SI Table S13).

It is also worth mentioning that the influences of religious compatibility on vaccine acceptance changed by age, which was remarkably stronger among older people (aged 45 or above) than the younger generations (SI Figure S3, SI Table S13). Additionally, when comparing the vaccine intention among people with the same attitudes towards the compatibility issue, the effects of age were only prominent for people supporting religious compatibility of vaccines (OR: 1.66, 95% CI: 1.44–1.89).

## Discussion

In this cross-sectional study, we examined the relationship between religious beliefs and perceptions of vaccines in various contexts characterized by diverse religious landscapes at different time points. Specifically, utilizing ~28,000 samples collected in two survey waves, we explored variations in attitudes towards religious compatibility and acceptance of the COVID-19 vaccines among subgroups of various religious affiliations in Africa and Asia Pacific over a one-year period from mid-2021 to 2022.

We found that Atheists, constituting a substantial population in the Asia Pacific region, displayed the highest degree of skepticism towards the compatibility of religions and vaccines. The low support rates, however, are not unexcepted, given that a majority of Atheists have no religious affiliations^24^, rendering this religion-related statement not applicable to them. Apart from this particular group, Buddhists in the Asia Pacific were the least likely to believe vaccines were compatible with their religious beliefs. Similar trends were observed among individuals of different demographic characteristics, including gender, age, and country of origin, in both waves of the surveys. These findings align with prior research, which reported significantly higher proportions of people having doubts about religious compatibility of vaccines in Southeast Asian and Western Pacific countries such as Thailand and Mongolia, where Atheism and Buddhism are dominant^12^.

We observed a decline in vaccine acceptance among Muslims between the two survey waves, as evidenced by reduced support for religious compatibility in both Africa and Asia Pacific (albeit marginal for African Muslims) and lower vaccine acceptance proportions in Africa during Wave 2. In addition, Muslims showed the mildest increase in vaccine intent among various religious groups in Asia Pacific (SI Table S11). The increase, nevertheless, was mainly driven by the rising vaccination rates, since only two out of the 24 unvaccinated respondents held a positive attitude towards the COVID-19 vaccines (SI Table S5). This phenomenon might be explained by the religious considerations regarding the permissibility of vaccines in Islam^25^. It is also worth mentioning that Muslims exhibited higher vaccine willingness during Wave 1 compared to Christians, which agrees with a previous finding of the negative association between Christianity and vaccination^13^. However, these patterns were not evident during Wave 2, partially because of the substantial increase in vaccine willingness among Christians in both regions (SI Table S11). Furthermore, we also found significantly lower vaccine willingness in Africa during both waves, potentially due to a combination of the late availability of COVID-19 vaccines and the prevailing vaccine hesitancy in this region^26^.

Our findings further showed interactions between faith and education. We spotted greater improvements in the support for the COVID-19 vaccines as well as perceived compatibility between faith and vaccines among people with lower education levels. This might be attributed to the mass vaccination campaigns that expanded the availability of vaccines for this subpopulation, who had previously been more likely to reject vaccination due to limited awareness of registration procedures^12^. Additionally, the rising prevalence of new, highly transmissible variants heightened concerns about COVID-19, motivating those who had initially been reluctant to seek vaccination to change their stance and get immunized^27^. While we observed more education contributed to more vaccine confidence in some religious groups, we also noticed heightened concerns regarding compatibility issues and increased vaccine hesitancy among Animists and Buddhists with higher education levels in Asia Pacific, which could possibly be explained by the group’s greater skepticism towards information disseminated by mainstream media sources^28^. Such mixed effects of education have also been identified in a previous study, but the negative association between education and vaccine confidence was claimed to be more pronounced in upper-middle or high-income countries^29^.

In addition, our study revealed a favorable impact of acknowledging compatibility between faith and vaccines on vaccine acceptance, which is consistent with a past analysis suggesting that individuals who endorsed religious compatibility of vaccines were twice as likely to receive a COVID-19 vaccine compared to those who did not support it^30^. The robustness of the association, however, was diminished during Wave 2 of the surveys. This variation might be ascribed to the widespread increase in vaccine acceptance across diverse populations due to improved vaccine availability, coupled with evolving social and political dynamics, as previously discussed.

It is also worth highlighting some of the innovative approaches employed in processing survey data, which facilitated between-group comparisons and allowed for a more meaningful analysis. These included the incorporation of sample weights in the regression models and the choice of a combined measure that encompassed willingness to accept the COVID-19 vaccines for the unvaccinated in addition to individual immunization status. The former approach enabled a better representation of the population of interest, while the latter took into account the potential disparity between intention and actual behavior regarding vaccination^31^ as well as the limited accessibility of the COVID-19 vaccines in certain African countries^26,32^, thus better characterizing people’s support of the COVID-19 vaccines.

Due to the restricted population size of certain religious groups in specific Asia Pacific countries, we pooled the data from different countries to infer the association between religious beliefs and perceptions of vaccines. This greatly inflated the sample sizes, mitigating the influences of potential outliers or random variations and thus better recovering the underlying relationships in the general population^33^. However, bias might be introduced by the possible differences in the distribution of people residing in different countries within each religious group across the two survey waves. This is because country-level factors, such as disease transmission patterns, vaccine-related policies implemented, and prevalence of misinformation, could also influence people’s attitudes towards vaccines^11,12,34^, but were not accounted for in our models. To address this, we distinguished between respondents from Africa and Asia Pacific, even if they shared the same religious beliefs, which enabled us to incorporate regional and racial influences into our models given the specific ethnic compositions in these regions. Neither did we consider the impacts of employment or socio-economic status, two other potential contributors to vaccine intent and access^18,35,36^, due to limited data access, although the inclusion of education level may have mitigated the impact of this limitation.

One additional limitation of our study pertains to the high acceptance rates in Asia Pacific during survey Wave 2, particularly for Animists and Hindus, among whom only one and three respondents rejected the COVID-19 vaccines, respectively. Such a substantial class imbalance posed substantial challenges in accurately assessing the effects of individual covariates. Meanwhile, potential confounders, such as increased public awareness of infection risks, further added intricacy to this issue^37,38^. An alternative approach could involve considering acceptance of a booster dose as the measure for vaccine acceptance, but booster hesitancy might differ from hesitancy towards the primary vaccination series^11^, making it challenging to quantify variations in vaccine sentiments across different years.

A further limitation is the timing discrepancies between the two rounds of the surveys carried out in Africa and Asia Pacific. This may have contributed to the smaller changes in attitudes towards vaccines observed among Africans, since the time intervals between the two survey waves were shorter in Africa compared to those in Asia Pacific and might not have coincided with the mass COVID-19 vaccination campaigns, when people’s sentiments towards vaccines were most likely to evolve^39^. To address this constraint, in the main analysis, we classified people residing in different regions into distinct religious groups, despite their shared religious beliefs, while we also conducted another sensitivity analysis by substituting the original binary time variable with a continuous one representing the exact time when the surveys were conducted (Supplementary Data 3).

Despite the limitations, our study sheds light on the connection between religious beliefs and vaccine sentiments, and additionally, how it was influenced by education in Africa and Asia Pacific in the context of COVID-19 vaccination programs. Our findings support the strong association between faith and vaccine acceptance, while the disparities among various religious groups substantiate the notion that the impact of religious beliefs on vaccine attitudes is often context-specific and thus should not be analyzed separately^12,18^. Moreover, the inconsistent patterns observed at different time points underscore the importance of longitudinal monitoring of vaccine sentiments among the population.

The COVID-19 pandemic, although no longer categorized as a global health emergency^40^, continues to pose high risks to countries worldwide due to the ongoing mutation of the virus. Given that neither infection nor vaccination can provide lifelong immunity, administration of booster doses is necessary to enhance protection alongside initial vaccination^11^. Further studies should, therefore, prioritize exploring latent factors driving the evolution of vaccine willingness over time and how their effects vary by region, faith, educational background, or other socio-demographic characteristics. The investigations should utilize longitudinal data on vaccine confidence spanning a greater time scale, with greater emphasis on understanding the dynamics of these potential influencers. Such comprehensive analyses will yield deeper insights into contributors to vaccine hesitancy, including their temporal variations, thereby facilitating the formulation of religion-tailored strategies that enhance immunization coverage and reduce disease burden^26^.

## Supplementary information

Supplementary Information

Description of Additional Supplementary Files

Supplementary Data 1

Supplementary Data 2

Supplementary Data 3

Supplementary Data 4

Supplementary Data 5

Supplementary Data 6

Supplementary Data 7

Supplementary Data 8

## Data Availability

The datasets used and analyzed during the current study are available from the corresponding author on reasonable request. Source data for the five figures in the manuscript are available in Supplementary Data 4–8. All the Supplementary Data can be accessed through https://github.com/ShihuiJin/vaccine_survey_outputs.

## Code availability

The underlying code for this study is not publicly available but may be made available to qualified researchers on reasonable request from the corresponding author.

## References

[1] COVID-19 Vaccines with WHO Emergency Use Listing. WHO - prequalification of medical products (IVDs, medicines, vaccines and immunization devices, vector control) https://extranet.who.int/pqweb/vaccines/vaccinescovid-19-vaccine-eul-issued (2021).

[2] de Figueiredo, A., Simas, C. & Larson, H. J. COVID-19 vaccine acceptance and its socio-demographic and emotional determinants: a multi-country cross-sectional study. *Vaccine* **41**, 354–364 (2023). 36414475 10.1016/j.vaccine.2022.10.051 PMC9647027

[3] Watson, O. J. et al. Global impact of the first year of COVID-19 vaccination: a mathematical modelling study. *Lancet Infect. Dis.* **22**, 1293–1302 (2022). 35753318 10.1016/S1473-3099(22)00320-6 PMC9225255

[4] Wu, N. et al. Long-term effectiveness of COVID-19 vaccines against infections, hospitalisations, and mortality in adults: findings from a rapid living systematic evidence synthesis and meta-analysis up to December, 2022. *Lancet Respir. Med.* **11**, 439–452 (2023). 36780914 10.1016/S2213-2600(23)00015-2 PMC9917454

[5] Hale, T. et al. A global panel database of pandemic policies (Oxford COVID-19 government response tracker). *Nat. Hum. Behav.* **5**, 529–538 (2021). 33686204 10.1038/s41562-021-01079-8

[6] Rydland, H. T., Friedman, J., Stringhini, S., Link, B. G. & Eikemo, T. A. The radically unequal distribution of Covid-19 vaccinations: a predictable yet avoidable symptom of the fundamental causes of inequality. *Humanit. Soc. Sci. Commun.* **9**, 1–6 (2022).

[7] Share of people who received at least one dose of COVID-19 vaccine. *Our World in data* https://ourworldindata.org/grapher/share-people-vaccinated-covid. (2024).

[8] WDI - The World by Income and Region. https://datatopics.worldbank.org/world-development-indicators/the-world-by-income-and-region.html. (2023).

[9] MacDonald, N. E. Vaccine hesitancy: definition, scope and determinants. *Vaccine* **33**, 4161–4164 (2015). 25896383 10.1016/j.vaccine.2015.04.036

[10] Larson, H. J. Defining and measuring vaccine hesitancy. *Nat. Hum. Behav.* **6**, 1609–1610 (2022). 36418535 10.1038/s41562-022-01484-7 PMC9684976

[11] Lazarus, J. V. et al. A survey of COVID-19 vaccine acceptance across 23 countries in 2022. *Nat. Med.* **29**, 366–375 (2023). 36624316 10.1038/s41591-022-02185-4

[12] Larson, H. J. et al. The state of vaccine confidence 2016: global insights through a 67-country survey. *EBioMedicine* **12**, 295–301 (2016). 27658738 10.1016/j.ebiom.2016.08.042 PMC5078590

[13] Trepanowski, R. & Drążkowski, D. Cross-national comparison of religion as a predictor of COVID-19 vaccination rates. *J. Relig. Health* **61**, 2198–2211 (2022). 35556198 10.1007/s10943-022-01569-7 PMC9095816

[14] Wong, L. P., Alias, H., Wong, P.-F., Lee, H. Y. & AbuBakar, S. The use of the health belief model to assess predictors of intent to receive the COVID-19 vaccine and willingness to pay. *Hum. Vaccines Immunother.* **16**, 2204–2214 (2020). 10.1080/21645515.2020.1790279 PMC7553708 32730103

[15] Olagoke, A. A., Olagoke, O. O. & Hughes, A. M. Intention to vaccinate against the novel 2019 coronavirus disease: the role of health locus of control and religiosity. *J. Relig. Health* **60**, 65–80 (2021). 10.1007/s10943-020-01090-9 PMC7596314 33125543

[16] Corcoran, K. E., Scheitle, C. P. & DiGregorio, B. D. Christian nationalism and COVID-19 vaccine hesitancy and uptake. *Vaccine* **39**, 6614–6621 (2021). 34629205 10.1016/j.vaccine.2021.09.074 PMC8489517

[17] Milligan, M. A., Hoyt, D. L., Gold, A. K., Hiserodt, M. & Otto, M. W. COVID-19 vaccine acceptance: influential roles of political party and religiosity. *Psychol. Health Med.* **27**, 1907–1917 (2022). 34407721 10.1080/13548506.2021.1969026

[18] Costa, J. C., Weber, A. M., Darmstadt, G. L., Abdalla, S. & Victora, C. G. Religious affiliation and immunization coverage in 15 countries in Sub-Saharan Africa. *Vaccine* **38**, 1160–1169 (2020). 31791811 10.1016/j.vaccine.2019.11.024 PMC6995994

[19] Pew Research Center. The global religious landscape. *Pew research center’s religion & public life project* https://www.pewresearch.org/religion/2012/12/18/global-religious-landscape-exec/ (2012).

[20] The Vaccine Confidence Project. https://www.vaccineconfidence.org.

[21] R Core Team. R: a language and environment for statistical computing. R Foundation for Statistical Computing (2024).

[22] Lumley, T., Gao, P. & Schneider, B. Survey: analysis of complex survey samples. (2024).

[23] Paul Murrell. The grid graphics package. (2024).

[24] Cragun, R. T. Nonreligion and Atheism. In: *Handbook of Religion and Society* (ed. Yamane, D.) 301–320 10.1007/978-3-319-31395-5_16. (Springer International Publishing, Cham, 2016).

[25] Syed Alwi, S. A. R. et al. A survey on COVID-19 vaccine acceptance and concern among Malaysians. *BMC Public Health* **21**, 1129 (2021). 34118897 10.1186/s12889-021-11071-6 PMC8196915

[26] Mutombo, P. N. et al. COVID-19 vaccine hesitancy in Africa: a call to action. *Lancet Glob. Health* **10**, e320–e321 (2022). 34942117 10.1016/S2214-109X(21)00563-5 PMC8687664

[27] Scrima, F., Miceli, S., Caci, B. & Cardaci, M. The relationship between fear of COVID-19 and intention to get vaccinated. The serial mediation roles of existential anxiety and conspiracy beliefs. *Pers. Individ. Differ.* **184**, 111188 (2022). 10.1016/j.paid.2021.111188 PMC8354796 34393312

[28] Jafar, A. et al. COVID-19 vaccine hesitancy in Malaysia: exploring factors and identifying highly vulnerable groups. *PLoS One* **17**, e0270868 (2022). 35802652 10.1371/journal.pone.0270868 PMC9269452

[29] Bergen, N. et al. Global state of education-related inequality in COVID-19 vaccine coverage, structural barriers, vaccine hesitancy, and vaccine refusal: findings from the Global COVID-19 trends and impact survey. *Lancet Glob. Health* **11**, e207–e217 (2023). 36565702 10.1016/S2214-109X(22)00520-4 PMC9771421

[30] Oduwole, E. O., Esterhuizen, T. M., Mahomed, H. & Wiysonge, C. S. Estimating vaccine confidence levels among healthcare staff and students of a tertiary institution in South Africa. *Vaccines* **9**, 1246 (2021). 34835177 10.3390/vaccines9111246 PMC8618030

[31] Shiloh, S., Peleg, S. & Nudelman, G. Vaccination against COVID-19: a longitudinal trans-theoretical study to determine factors that predict intentions and behavior. *Ann. Behav. Med.* **56**, 357–367 (2022). 34864833 10.1093/abm/kaab101

[32] Hassan, M. A.-K. & Aliyu, S. Delayed access to COVID-19 vaccines: a perspective on low-income countries in Africa. *Int. J. Health Serv.* **52**, 323–329 (2022). 35469499 10.1177/00207314221096365 PMC9047600

[33] Nemes, S., Jonasson, J. M., Genell, A. & Steineck, G. Bias in odds ratios by logistic regression modelling and sample size. *BMC Med. Res. Methodol.* **9**, 56 (2009). 19635144 10.1186/1471-2288-9-56 PMC2724427

[34] Kim, K., Lee, C., Ihm, J. & Kim, Y. A comprehensive examination of association between belief in vaccine misinformation and vaccination intention in the COVID-19 context. *J. Health Commun.* **27**, 495–509 (2022). 36205037 10.1080/10810730.2022.2130479

[35] Hara, M., Ishibashi, M., Nakane, A., Nakano, T. & Hirota, Y. Differences in COVID-19 vaccine acceptance, hesitancy, and confidence between healthcare workers and the general population in Japan. *Vaccines* **9**, 1389 (2021). 34960135 10.3390/vaccines9121389 PMC8707052

[36] Wong, L. P. et al. Acceptability for COVID-19 vaccination: perspectives from Muslims. *Hum. Vaccines Immunother.* **18**, 2045855 (2022). 10.1080/21645515.2022.2045855 PMC9196781 35439106

[37] İkiışık, H., Akif Sezerol, M., Taşçı, Y. & Maral, I. COVID-19 vaccine hesitancy: a community-based research in Turkey. *Int. J. Clin. Pract.* **75**, e14336 (2021). 33973322 10.1111/ijcp.14336 PMC8237055

[38] Saito, K., Komasawa, M., Aung, M. N. & Khin, E. T. COVID-19 vaccination willingness in Four Asian Countries: a comparative study including Thailand, Indonesia, the Philippines, and Vietnam. *Int. J. Environ. Res. Public. Health* **19**, 12284 (2022). 36231586 10.3390/ijerph191912284 PMC9566518

[39] Nomura, S. et al. Characterising reasons for reversals of COVID-19 vaccination hesitancy among Japanese people: one-year follow-up survey. *Lancet Reg. Health West. Pac.* **27**, 100541 (2022). 35892010 10.1016/j.lanwpc.2022.100541 PMC9302916

[40] WHO. Statement on the fifteenth meeting of the IHR (2005). Emergency Committee on the COVID-19 pandemic. https://www.who.int/news/item/05-05-2023-statement-on-the-fifteenth-meeting-of-the-international-health-regulations-(2005)-emergency-committee-regarding-the-coronavirus-disease-(covid-19)-pandemic (2023).

