# Supplementary material for: Comparing role of religion in perception of the COVID-19 vaccines in Africa and Asia Pacific
Source: Commun Med (Lond). 2024 Oct 24;4:212. doi: 10.1038/s43856-024-00628-2 (PMC11502740; doi:10.1038/s43856-024-00628-2)
Supplement: Supplementary file 1 — Supplementary Information [file 43856_2024_628_MOESM1_ESM.pdf]

Supplementary information

1. Survey information 2
2. Summary of respondents belonging to different subpopulations 6
3. Selection of the survey questions for analysis 7
4. Quantifying the heterogenous impacts of religions 8
References 16

1. Survey information

Sampling approach: Samples in all countries consisted of adults (over 18 years old) residing in the survey countries and speaking at least one of the survey languages. The final samples were supposed to be representative of the wider population in terms of age and gender, and weights would be assigned if the final sample differed significantly from the available national data. More specifically, in CATI (computer-assisted telephonic interviews) countries, the sample was chosen from existing telephone databases of respondents who have registered to take part in opinion surveys, and quotas were set to ensure an appropriate distribution in terms of gender, age, and region. In countries where surveys were conducted online, a similar approach was used where a sample was drawn from existing online panels with quotas set to ensure a representative spread of the population. In countries where CAPI (computer-assisted personal interviews) or F2F (face to face) methodology was used, the number of interviews to be conducted in each region was determined by the distribution of the population according to the latest estimates, after which an appropriate number of Primary Sampling Units (PSUs, specific areas in which interviews are to be conducted) were randomly selected from the best available database and interviewing teams were then dispatched to the required areas to conduct the interviews.

Respondent selection: For in-person interviews, the interviewing teams, which were composed of interviewers and a supervisor, selected a local focal point as the starting point at the required PSUs. Then, each interviewer proceeded in a different direction, utilizing a randomizing 'skip pattern' derived from the day's date to select households and establish contact with residents. After contact was made, a respondent within each household was randomly chosen using a Kish Grid approach and interviewers would either conduct the interview with him or her or schedule a call-back appointment. The interview would be terminated immediately once the respondent withdrew his or her consent to interview.

Languages: The original survey was composed in English by ORB International, but was translated by local fieldwork partners and verified by ORB staff.

Quality control: Before fieldwork started, all interviewers were trained on the questionnaire, while all survey scripts (CATI, CAPI and F2F) underwent thorough checks to ensure all appropriate logic checks were in place. In CATI countries, interviewers' first calls were overseen by a supervisor to ensure their use of proper interviewing techniques. The duration of interview was also monitored to prevent the interviewers from speeding, in terms of the length for both the entire interview and specific sections within the survey. Between 10% and 15% of calls per country were randomly selected for back-checking to ensure validity of data. Meanwhile, all face-to-face interviews were closely monitored during fieldwork through daily checking of new interviews, assessing duration of the overall interview and specific sections within the survey, verification of interview location accuracy via GPS, and review of interviewer recordings to ensure correct methodology was followed. Upon completion of fieldwork in each country, the data were 'cleaned' to ensure a comprehensive and reliable final dataset.

Table S1. Summary of the sampling approaches, languages, and sample sizes of the two surveys in each of the 14 countries in Africa and Asia Pacific. CATI means computer-assisted telephonic interviews, CAPI means computer-assisted personal interviews, and F2F means face-to-face interviews. A mixed CATI and CAPI approach was used in Vietnam in Wave 1 to achieve the 1000 samples. Surveys for Malaysia in Wave 2 were in either Malay or English.

Region	Country	Sampling Approach	Languages	Sample size (Wave 1)	Sample size (Wave 2)
Africa	**Cameroon**	F2F	French	1,058	1,008
	**DRC**	F2F	French	1,139	1,021
	**Kenya**	F2F	English, Swahili/Kiswahili	1,024	1,078
	**Nigeria**	F2F	English, Hausa, Yoruba	1,030	1,008
	**Senegal**	F2F	French, Wolof, Pular	1,152	1,001
	**South Africa**	F2F	English	1,032	982
	**Uganda**	F2F	English, Luo, Luganda, Lumabasa, Runyankole	1,022	1,081
Asia Pacific	**Cambodia**	CATI	Khmer	1,000	1,000
	**Japan**	Online	Japanese	1,044	1,066
	**Laos**	CATI	Lao	1,000	1,000
	**Malaysia**	CATI	Malay (Waves 1 & 2), English (Wave 2)	1,000	1,000
	**Mongolia**	CATI	Mongolian	1,000	1,000
	**Philippines**	CATI	Tagalog, Hiligaynon, Cebuano	1,000	1,000
	**South Korea**	Online	Korean	1,133	1,155
	**Vietnam**	CAPI (Wave 2), CATI	Vietnamese	1,044	1,003

Table S2. Time of the two surveys for Wave 1 and Wave 2 in each of the 14 countries in Africa and Asia Pacific. Dates are in dd/mm/yyyy format.

Region	Country	Wave 1	Wave 2
Africa	**Cameroon**	25/01/2022–22/02/2022	21/07/2022–30/08/2022
	**DRC**	03/02/2022–01/03/2022	26/07/2022–11/08/2022
	**Kenya**	14/01/2022–24/01/2022	14/07/2022–31/07/2022
	**Nigeria**	25/01/2022–08/02/2022	01/08/2022–31/08/2022
	**Senegal**	21/01/2022–27/02/2022	27/07/2022–26/08/2022
	**South Africa**	17/01/2022–03/03/2022	07/07/2022–31/08/2022
	**Uganda**	10/01/2022–30/01/2022	18/06/2022–20/08/2022
Asia Pacific	**Cambodia**	16/06/2021–04/07/2021	17/05/2022–26/05/2022
	**Japan**	05/07/2021–08/07/2021	17/05/2022–26/05/2022

**Laos**	17/06/2021–04/07/2021	17/05/2022–26/05/2022
**Malaysia**	14/06/2021–27/06/2021	20/05/2022–20/06/2022
**Mongolia**	22/06/2021–30/06/2021	01/05/2022–15/05/2022
**Philippines**	11/06/2021–04/07/2021	12/05/2022–31/05/2022
**South Korea**	05/07/2021–14/07/2021	19/05/2022–01/06/2022
**Vietnam**	16/06/2021–14/07/2021	17/05/2022–26/05/2022

Contents of the surveys

For surveys conducted in Asia Pacific, questions were mainly related to the following four topics: socio-demographics, media landscape, knowledge and attitudes towards COVID-19 and vaccination, confidence for vaccines in general and COVID-19 related rumour management, while in Africa, an additional section named media landscape was included, which asked the respondents their ways to access information. Responses to most of the questions are either yes or no, or on a Likert scale, and the respondents could choose the answer that best matched their perceptions. We did not include any open-ended questions in our analyses. Respondents were supposed to answer all the survey questions, but they might choose “Don’t know” or “Refused to say” if they did not wish to provide an answer. The surveys were conducted independently for Wave 1 and 2. In Africa, the survey time mostly fell in early and mid 2022, while in Asia Pacific, the time for the two waves was mid 2021 and 2022 respectively.

Table S3. Proportion (%) of missing or invalid information in survey responses, categorized by region and survey wave. ‘Overall’ denotes the proportion of respondents belonging to the selected religious groups but excluded from our analysis due to missing or invalid responses.

	Age	Gender	Education	Compatibility	Overall
**Africa, Wave 1**	0.01	0	0.3	0.7	1.0
**Asia Pacific, Wave 1**	0	0.5	0.7	0	1.0
**Africa, Wave 2**	0.1	0.01	0.5	0.2	0.8
**Asia Pacific, Wave 2**	0	0.6	0.8	0	1.2

Additional background information

We estimated the mean vaccination rates at the time of the surveys using statistics from Our World in Data [1], whose data are from public official sources only. Since not all the countries reported their vaccination data on a daily basis, we interpolated and extrapolated the missing parts using ‘gam’ function in R package `mgcv` [2], and took the average over the entire survey periods. Generally, the vaccination rates are lower in the African countries compared to those in Asia Pacific. Note that in our analyses, we focused on adults accepting the COVID-19 vaccines, which would naturally be higher than the actual vaccination rates, since in such countries as the Philippines and South Korea, vaccines might not be universally accessible by the time of the surveys, though they were all free of charge [3].

Table S4. Summary of vaccination rates (%, proportion of people receiving at least one dose) **in each country at the time of the surveys.**

Region	Country	Wave 1	Wave 2
**Africa**	**Cameroon**	3.8	5.4
	**DRC**	11	12
	**Kenya**	11	24
	**Nigeria**	6.5	18
	**Senegal**	8.2	8.4
	**South Africa**	33	37
	**Uganda**	23	37
**Asia Pacific**	**Cambodia**	23	89
	**Japan**	31	83
	**Laos**	11	77
	**Malaysia**	16	83
	**Mongolia**	61	65
	**Philippines**	5.7	65
	**South Korea**	30	87
	**Vietnam**	3.0	85

2. Summary of respondents belonging to different subpopulations

We summarized the respondents in each wave by religious beliefs and region (henceforth referred to as 'religious groups') as Supplementary Data 1. In total, there were eight distinct religious groups. Sample sizes for the groups were unbalanced in each wave of the surveys. Over half of the African respondents were Christians, while Buddhists were the largest group in Asia Pacific. The original five age groups (18–24, 25–34, 35–44, 45–54, 55+) and seven education levels (no formal education, primary, secondary, vocational, university, Master/PhD, other) were respectively merged into two, since the original subgroup populations for some religions (e.g., Hinduism in Asia Pacific) might be too small (<5) for a meaningful analysis and neighbouring groups often responded similarly to the survey questions of interest. In this way, all the socio-demographic factors included in our study but religion—age, gender, education—were binary.

Table S5. Estimated proportion (%; mean and 95% confidence interval [CI]) of respondents having been vaccinated (among the total subpopulation) or accepting the COVID-19 vaccines (among the unvaccinated or individuals with unknown vaccination status) for different religious groups during Wave 1 and Wave 2 of the surveys. AP stands for Asia Pacific.

	Vaccination (Wave 1)	Vaccination (Wave 2)	Acceptance (Wave 1)	Acceptance (Wave 2)
**Christian (Africa)**	37.5 [36.1,38.8]	48.2 [46.7,49.6]	69.9 [68.2,71.5]	68.6 [66.8,70.4]
**Muslim (Africa)**	34.8 [32.8,36.9]	37.0 [34.9,39.2]	74.3 [71.9,76.6]	68.2 [65.6,70.8]
**Animist (AP)**		98.4 [95.7,99.4]	96.4 [93.2,98.1]	74.7 [3.8,99.6]
**Atheist (AP)**		95.5 [94.3, 96.5]	96.0 [94.9, 96.9]	28.4 [18.5,41.0]
**Buddhist (AP)**		97.2 [96.6,97.7]	94.8 [94,95.5]	39.3 [29.8,49.7]
**Christian (AP)**		92.6 [91.1,93.8]	87.8 [86,89.3]	52.8 [43.2,62.1]
**Hindu (AP)**		96.9 [90.5,99.0]	87.3 [77.1,93.4]	0 [0,0]
**Muslim (AP)**		96.0 [94.1,97.3]	92.5 [90.1,94.3]	8.3 [1.9,30.2]

3. Selection of the survey questions for analysis

We assessed the respondents' attitudes towards vaccines through their perceptions towards religious compatibility of vaccines ("religious compatibility" or "compatibility" in short) and acceptance of the COVID-19 vaccines ("acceptance" in short). Compatibility was associated with the statement "Vaccines are compatible with my religious beliefs". Those who gave a positive response (i.e., choosing "tend to agree" or "strongly agree") were supposed to supporters, while those with a non-positive answer, including "tend to disagree", "strongly disagree" and "don't know", were taken as opponents to religious compatibility of vaccines. As for acceptance, in the surveys all but respondents in Asia Pacific in Wave 1 were asked whether they had been vaccinated and further questions regarding the willingness to accept the COVID-19 vaccines to protect oneself, family, friends or people at risk were asked to the unvaccinated or those who did not provided their vaccination status. Therefore, we only take the unvaccinated who were reluctant to accept the COVID-19 vaccines for anyone among themselves, their families, friends, and people at risk, together with those who refused to answer questions related to vaccine acceptance, as the group rejecting the COVID-19 vaccines (Table S5).

Table S6. Survey questions of interest and how responses in the raw data corresponded to the recoded numeric values used in analyses.

	Related Questions/statements	Recoded response: 0	Recoded response: 1
**Compatibility**	Vaccines are compatible with my religious beliefs.	"Strongly disagree"; "Tend to disagree"; "Don't know".	"Strongly agree", "Tend to agree"
**Acceptance**	Have you been vaccinated against COVID-19?	"No"; "Don't know/ Refused".	"Yes—I have had one dose"; "Yes—I have had two doses"; "Yes—I have had an additional or booster dose".
	Would you accept a COVID-19 vaccine to protect myself /family /friends / at-risk groups?*	"Definitely no"; "Unsure but leaning towards no"; "Don't know/ Refused".	"Definitely yes"; "Unsure but leaning towards yes".

*This question was only asked to the unvaccinated or residents in Asia Pacific in 2021.

4. Quantifying the heterogenous impacts of religions

Table S7. Information for factors included in the logistic regression models.

Factor name	Levels	Baseline for regression models
**Religion (Religious group)**	Christian (Africa), Muslim (Africa), Animist (AP), Atheist (AP), Buddhist(AP), Christian (AP), Hindu (AP), Muslim (AP)	Christian (Africa)
**Age**	18–44, 45+	18–44
**Gender**	Female, Male	Female
**Education**	Secondary or below, Post-secondary	Secondary or below
**Time**	Wave 1, Wave 2	Wave 1

Proportions of people supporting religious compatibility of vaccines, the COVID-19 vaccine acceptance rates, and the coefficients for the three logistic regression models were estimated using the `survey` [4] package in R [5].

Selection of the interaction terms for the regression models

Assuming effects of religions varied by time, age, gender and education levels, we started with the full model which predicted one's probability of supporting religious compatibility or accepting the COVID-19 vaccines with interaction terms between religion (short for 'religion by region' or 'religious group'), time and any one among age, gender and education (i.e., $\text{religion} \times \text{time} \times \text{age} + \text{religion} \times \text{time} \times \text{gender} + \text{religion} \times \text{time} \times \text{education}$) and subsequently dropped the interactions with *age* or *gender* after observing that the estimated coefficients for these terms were all insignificant. However, since we also noticed remarkable differences in effects of age between the two genders (or equivalently, effects of gender between the two age groups), we included an interaction term between age and gender.

As for the model wherein we included *religious compatibility* as an explanatory variable for *COVID-19 vaccine acceptance*, on the basis of the final model of Acceptance, we added an interaction term between compatibility and all the other variables, assuming these factors' influences on the odds of accepting the COVID-19 vaccines varied by perception towards religious compatibility. However, we only found the estimated coefficients for interaction between religion, time, and compatibility were statistically significant and thus decided to keep this interaction term for our final model of Acceptance with Compatibility.

Table S8. Estimated proportion (%; mean and 95% confidence interval [CI]) of people feeling religious compatibility / accepting COVID-19 vaccines among different religious groups during Wave 1 and Wave 2 of the surveys. AP stands for Asia Pacific.

	Compatibility (Wave 1)	Compatibility (Wave 2)	Acceptance (Wave 1)	Acceptance (Wave 2)
**Christian (Africa)**	64.7 [63.3,66.0]	66.8 [65.4,68.1]	81.1 [80.0,82.2]	83.7 [82.7,84.7]
**Muslim (Africa)**	77.3 [75.5,79.1]	76.6 [74.7,78.5]	83.2 [81.6,84.8]	80.0 [78.2,81.7]
**Animist (AP)**	66.3 [60.1,71.9]	75.2 [69.1,80.4]	96.4 [93.2,98.1]	99.6 [97.2,99.9]
**Atheist (AP)**	45.1 [42.6, 47.6]	48.2 [45.2,51.2]	96.0 [94.9,96.9]	96.8 [95.7, 97.6]
**Buddhist (AP)**	49.0 [47.4,50.7]	56.1 [54.3,57.8]	94.8 [94.0,95.5]	98.3 [97.8,98.7]
**Christian (AP)**	67.8 [65.3,70.1]	76.6 [74.3,78.7]	87.8 [86.0,89.3]	96.5 [95.4,97.3]
**Hindu (AP)**	63.4 [51.3,74.0]	64.4 [54.2,73.5]	87.3 [77.1,93.4]	96.9 [90.5,99.0]
**Muslim (AP)**	74.4 [70.8,77.6]	64.2 [60.3,67.9]	92.5 [90.1,94.3]	96.4 [94.5,97.6]

Table S9. Pearson correlation between religious compatibility and COVID-19 vaccine acceptance. All are statistically significant at significance level of 0.10. **AP** stands for Asia Pacific.

	Wave 1	Wave 2
**Christian (Africa)**	0.17	0.12
**Muslim (Africa)**	0.16	0.10
**Animist (AP)**	0.18	0.11
**Atheist (AP)**	0.12	0.14
**Buddhist (AP)**	0.10	0.09
**Christian (AP)**	0.24	0.14
**Hindu (AP)**	0.24*	0.24
**Muslim (AP)**	0.22	0.22

* Insignificant at significance level of 0.05 (p-value = 0.07).

Table S10. Estimated log odds (mean and 95% CIs) for the three multivariate logistic regression models (i–iii). *Time*, *Edu*, *Age,* and *Compatible* are binary variables which equal 1 if the sample was collected during Wave 2 of the surveys, had received post-secondary education, aged 45 years old or above, and agreed with compatibility between vaccine and their religious beliefs, respectively. **AP** stands for Asia Pacific.

	Compatibility	Acceptance	Acceptance with Compatibility

**Time**	0.15***<br>[0.10,0.20]	0.28***<br>[0.20,0.35]	0.26***<br>[0.18,0.34]
**Muslim (Africa)**	0.55***<br>[0.47,0.64]	-0.04<br>[-0.14,0.05]	-0.17***<br>[-0.27,-0.07]
**Animist (AP)**	0.23**<br>[0.03,0.43]	2.34***<br>[1.72,2.97]	2.33***<br>[1.71,2.96]
**Atheist (AP)**	-0.83***<br>[-0.92,-0.74]	1.72***<br>[1.52,1.92]	1.94***<br>[1.74,2.14]
**Buddhist (AP)**	-0.59***<br>[-0.65,-0.52]	1.78***<br>[1.64,1.92]	1.95***<br>[1.81,2.09]
**Christian (AP)**	0.27***<br>[0.18,0.36]	0.90***<br>[0.75,1.04]	0.86***<br>[0.72,1.01]
**Hindu (AP)**	-0.05<br>[-0.37,0.27]	0.99***<br>[0.40,1.57]	1.03***<br>[0.44,1.61]
**Muslim (AP)**	0.19***<br>[0.06,0.32]	1.27***<br>[1.02,1.52]	1.25***<br>[1.01,1.50]
**Edu**	-0.05*<br>[-0.10,0]	0.02<br>[-0.06,0.11]	0.02<br>[-0.06,0.10]
**Male**	0.03<br>[-0.02,0.08]	0.07*<br>[-0.01,0.14]	0.07*<br>[-0.01,0.15]
**Age**	0.21***<br>[0.15,0.26]	0.07<br>[-0.03,0.16]	0.02<br>[-0.07,0.11]
**Compatible**			0.93***<br>[0.85,1.01]

* p-value < 0.10
** p-value < 0.05
*** p-value < 0.01

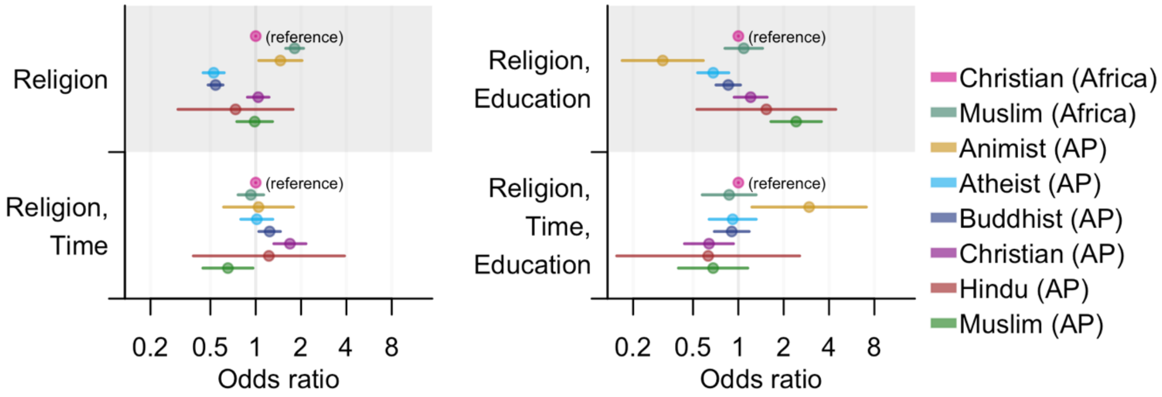

Figure S1. Estimated odds ratio for religion-related terms in Model Compatibility. Factors in an interaction are separated by commas. Religion means religion by region. AP stands for Asia Pacific.

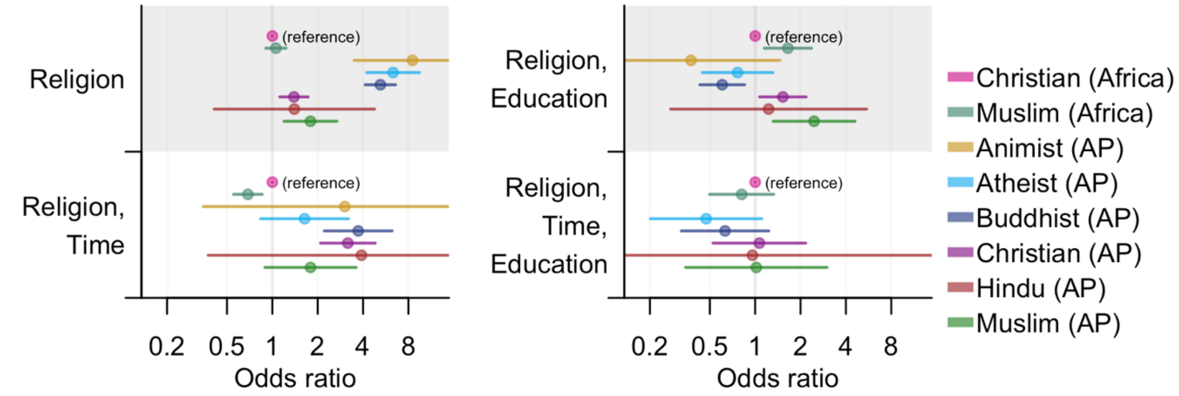

Figure S2. Estimated odds ratio for religion-related terms in Model Acceptance. Factors in an interaction are separated by commas. Religion means religion by region. AP stands for Asia Pacific.

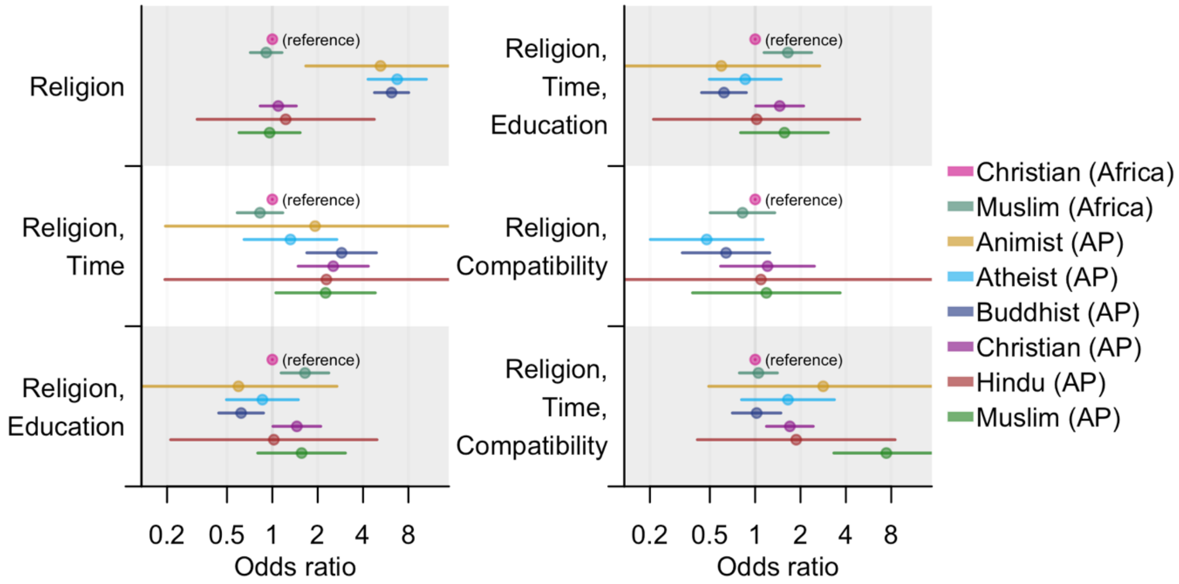

Figure S3. Estimated odds ratio for religion-related terms in Model Acceptance with Compatibility. Factors in an interaction are separated by commas. Religion means religion by region. **AP** stands for Asia Pacific.

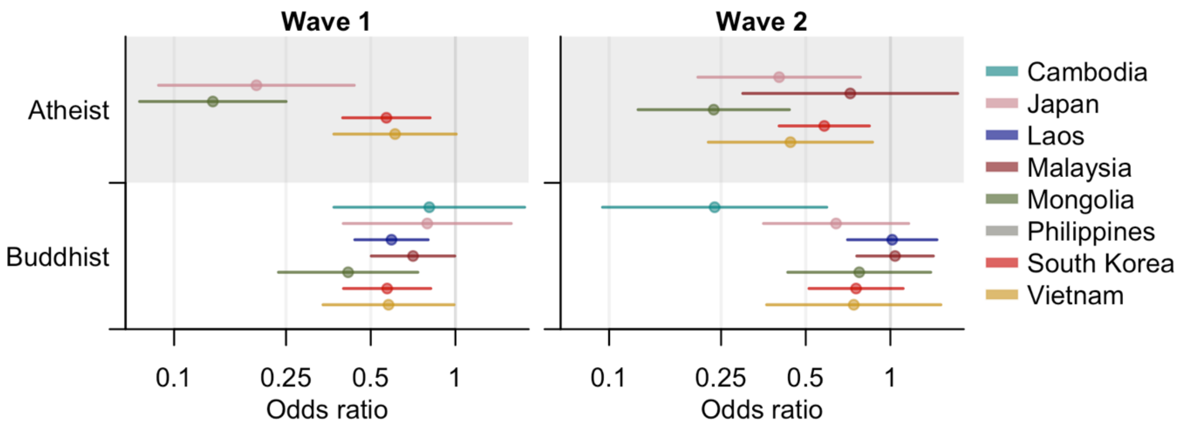

Figure S4. Estimated odds ratio for Atheists and Buddhists acknowledging compatibility between religions and vaccines in Asia-Pacific countries. The reference group consists of people believing in other religions—Atheists, Christians, Muslims, and Hindus. Only the estimates for religious groups with a size larger than 20 per survey are shown in the figure.

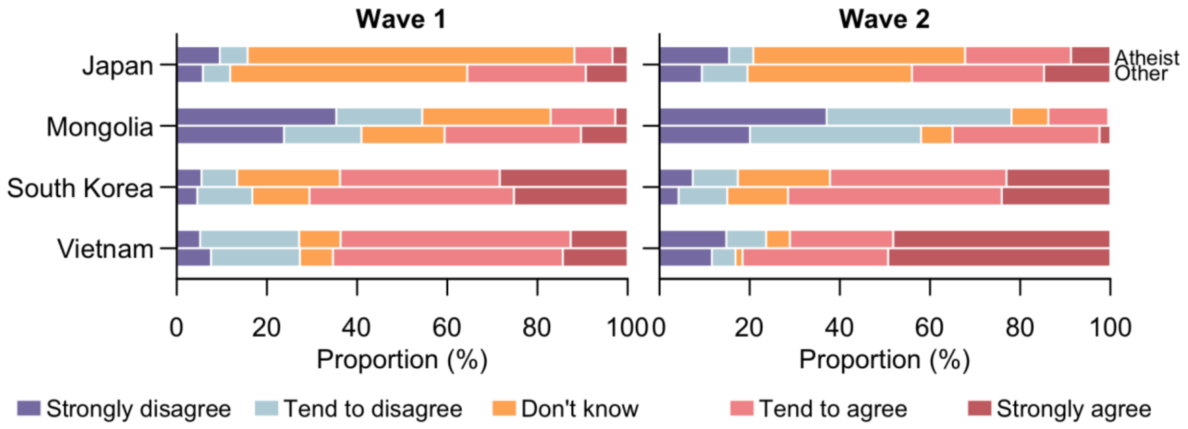

Figure S5. Response to the statement of compatibility between religions and vaccines in four Asia-Pacific countries (Japan, Mongolia, South Korea, and Vietnam). In these countries, over 50 Atheists participated in each of the two waves of surveys. Statistics for Atheist respondents are visualized in the row above while those for people believing in other religions are in the row below.

Table S11. Log odds (mean and 95% CI) of *Time* for supporting religious compatibility or accepting the COVID-19 vaccines in each religious group. Odds ratios are not used due to the large estimated coefficients for COVID-19 vaccine acceptance among Animists and Hindus in Asia Pacific in Wave 2. Almost all the respondents from these two groups accepted the COVID-19 vaccines. Effects presented in the table are the average across different subpopulations (i.e., people of different ages, genders, and with different education backgrounds). *Time* is a binary variable which equals 1 if the sample was collected during Wave 2 of the surveys. **AP** stands for Asia Pacific.

	**Compatibility**	**Acceptance**
**Christian (Africa)**	0.09** [0.01,0.18]	0.18*** [0.07,0.28]
**Muslim (Africa)**	-0.04 [-0.19,0.11]	-0.22** [-0.38,-0.05]
**Animist (AP)**	0.43** [0.03,0.83]	2.25** [0.17,4.33]
**Atheist (AP)**	0.12 [-0.03,0.28]	0.23 [-0.16,0.62]
**Buddhist (AP)**	0.28*** [0.18,0.38]	1.17*** [0.87,1.47]
**Christian (AP)**	0.44*** [0.28,0.61]	1.35*** [1.03,1.67]
**Hindu (AP)**	0.05 [-0.60,0.69]	1.50** [0.14,2.86]

**Muslim (AP)**	-0.48***<br>[-0.73,-0.24]	0.77***<br>[0.25,1.29]

* p-value < 0.10
** p-value < 0.05
*** p-value < 0.01

Table S12. Log odds (mean and 95% CI) of *Compatible* for accepting the COVID-19 vaccines in each religious group during each wave of the surveys. Odds ratios are not used due to the large estimated coefficients for Animists and Hindus in Asia Pacific in Wave 2. Almost all the respondents from these two groups accepted the COVID-19 vaccines. Acceptance means either the respondents had been vaccinated by the time of the survey or they were willing to accept the COVID-19 vaccines to protect themselves, their families, friends, or people at risk. Effects presented in the table are the average across different subpopulations (i.e., people of different ages, genders, and with different education backgrounds). *Compatible* is a binary variable which equals 1 if the respondent believed vaccines were compatible with their religious beliefs. **AP** stands for Asia Pacific.

	**Wave 1**	**Wave 2**
**Christian (Africa)**	0.85***<br>[0.70,1.00]	0.68***<br>[0.52,0.83]
**Muslim (Africa)**	0.90***<br>[0.65,1.15]	0.52***<br>[0.28,0.77]
**Animist (AP)**	2.00**<br>[0.40,3.61]	18.5***<br>[16.5,20.5]
**Atheist (AP)**	1.48***<br>[0.79,2.17]	2.33***<br>[1.30,3.37]
**Buddhist (AP)**	0.98***<br>[0.64,1.32]	1.45***<br>[0.84,2.05]
**Christian (AP)**	1.46***<br>[1.14,1.79]	1.45***<br>[0.88,2.02]
**Hindu (AP)**	1.44*<br>[-0.09,2.96]	19.2***<br>[18.0,20.5]
**Muslim (AP)**	2.91***<br>[2.12,3.70]	2.98***<br>[1.51,4.45]

* p-value < 0.10
** p-value < 0.05
*** p-value < 0.01

Table S13. Log odds (mean and 95% CI) of *Time* for accepting the COVID-19 vaccines in each religious group for people who disagreed and agreed with religious compatibility of vaccines respectively. Odds ratios are not used due to the large estimated coefficients for Animists and Hindus in Asia Pacific in Wave 2. Almost all the respondents from these two groups accepted the COVID-19 vaccines. Acceptance means either the respondents had been vaccinated by the time of the survey or they were willing to accept the COVID-19 vaccines to protect themselves, their families, friends, or people at risk. Effects presented in the table are the average across different subpopulations (i.e., people of different ages, genders, and with different education backgrounds). Incompatible and Compatible

respectively refer to the respondents who disagree and agree with compatibility between vaccines and their religious beliefs. **AP** stands for Asia Pacific.

	**Incompatible**	**Compatible**
**Christian (Africa)**	0.26***<br>[0.10,0.42]	0.09<br>[-0.06,0.23]
**Muslim (Africa)**	0.04<br>[-0.25,0.34]	-0.33***<br>[-0.53,-0.13]
**Animist (AP)**	1.73<br>[-0.42,3.88]	18.2***<br>[16.8,19.6]
**Atheist (AP)**	0.08<br>[-0.34,0.50]	0.93<br>[-0.24,2.10]
**Buddhist (AP)**	0.63***<br>[0.32,0.94]	1.44***<br>[0.84,2.04]
**Christian (AP)**	1.00***<br>[0.59,1.41]	1.24***<br>[0.75,1.72]
**Hindu (AP)**	0.34<br>[-0.97,1.64]	18.9***<br>[17.7,20.1]
**Muslim (AP)**	-0.18<br>[-0.65,0.29]	1.22<br>[-0.34,2.78]

* p-value < 0.10
** p-value < 0.05
*** p-value < 0.01

Table S14. Log odds (mean and 95% CI) of interaction terms between *Compatible* and *Religious groups* for accepting the COVID-19 vaccines during each wave of the surveys. Odds ratios are not used due to the large estimated coefficients for Animists and Hindus in Asia Pacific in Wave 2. Almost all the respondents from these two groups accepted the COVID-19 vaccines. The reference group is African Christians. Acceptance means either the respondents had been vaccinated by the time of the survey or they were willing to accept the COVID-19 vaccines to protect themselves, their families, friends, or people at risk. Effects presented in the table are the average across different subpopulations (i.e., people of different ages, genders, and with different education backgrounds). *Compatible* is a binary variable which equals 1 if the respondent believed vaccines were compatible with their religious beliefs. **AP** stands for Asia Pacific.

	**Wave 1**	**Wave 2**
**Muslim (Africa)**	0.05<br>[-0.24,0.34]	-0.15<br>[-0.45,0.14]
**Animist (AP)**	1.15<br>[-0.45,2.75]	11.8***<br>[9.78,13.8]
**Atheist (AP)**	0.63<br>[-0.07,1.33]	1.65***<br>[0.61,2.70]
**Buddhist (AP)**	0.13<br>[-0.24,0.50]	0.77**<br>[0.15,1.40]
**Christian (AP)**	0.61***<br>[0.25,0.97]	0.77**<br>[0.18,1.36]
**Hindu (AP)**	0.58<br>[-0.91,2.08]	13.6***<br>[12.3,14.8]

**Muslim (AP)**	2.06***<br>[1.26,2.86]	2.30***<br>[0.83,3.77]

* p-value < 0.10
** p-value < 0.05
*** p-value < 0.01

Sensitivity analysis: accounting for disparities in the survey time

We substituted the original binary variable Time with a continuous one to account for the disparities in the survey time for different countries. Specifically, we calculated the mean time (midpoint of the period) of each survey wave for each individual country and derived the value for *Time* as the difference (in year) between the mean time and the earliest survey time (i.e., that for Wave 1 in Malaysia). We repeated the analysis using the same logistic regression models as those in the main analysis. Estimation results using the models adjusted for survey times were similar to those in the main analysis in terms of statistical significance, but we also observed changes in the estimated coefficients for *Time* and *Education*, as well as the interaction between *Education* and *Christians in Asia Pacific* (Supplementary Data 3).

References

1. Share of people who received at least one dose of COVID-19 vaccine. *Our World in Data* https://ourworldindata.org/grapher/share-people-vaccinated-covid.
2. Wood, S. mgcv: Mixed GAM Computation Vehicle with Automatic Smoothness Estimation. (2022).
3. Hale, T. *et al.* A global panel database of pandemic policies (Oxford COVID-19 Government Response Tracker). *Nat. Hum. Behav.* **5**, 529–538 (2021).
4. Lumley, T. survey: Analysis of Complex Survey Samples. (2023).
5. R Core Team. R: A Language and Environment for Statistical Computing. R Foundation for Statistical Computing (2023).
