# Supplementary material for: Comparing role of religion in perception of the COVID-19 vaccines in Africa and Asia Pacific
Source: Commun Med (Lond). 2024 Oct 24;4:212. doi: 10.1038/s43856-024-00628-2 (PMC11502740; doi:10.1038/s43856-024-00628-2)
Supplement: Supplementary file 2 — Description of Additional Supplementary Files [file 43856_2024_628_MOESM2_ESM.pdf]

Description of Additional Supplementary Files

File name: Supplementary Data 1

File description: Summary of demographic characteristics (age, gender, education and country) of the respondents by religion for the two survey waves.

File name: Supplementary Data 2

File description: Estimated log odds (mean and 95% CIs) for the three multivariate logistic regression models with interactions (iv–vi).

File name: Supplementary Data 3

File description: Estimated log odds (mean and 95% CIs) for the three multivariate logistic regression models with interactions (iv– vi) in the sensitivity analysis.

File name: Supplementary Data 4–8

File description: raw data for Figure 1–5 in the main manuscript, respectively
